# The CpG island methylator phenotype defines an immune-cold and therapy-resistant subtype of cutaneous melanoma

**DOI:** 10.3389/fimmu.2026.1821360

**Published:** 2026-06-24

**Authors:** Wei Lu, Xiaowei Sha, Hua Lei, Chong Mao

**Affiliations:** Department of Dermatology, Sichuan Provincial People’s Hospital, University of Electronic Science and Technology of China, Chengdu, China

**Keywords:** CpG island methylator phenotype, cutaneous melanoma, DNA methylation, immunotherapy, tumor microenvironment

## Abstract

**Background:**

Cutaneous melanoma (CM) displays marked clinical heterogeneity and variable responses to immune checkpoint blockade (ICB). The CpG Island Methylator Phenotype (CIMP), characterized by widespread promoter hypermethylation, has been implicated in tumor progression, but its role in shaping the tumor immune microenvironment and therapeutic response remains unclear.

**Methods:**

DNA methylation and transcriptomic data from The Cancer Genome Atlas (TCGA) were analyzed and validated in an external cohort (GSE120878). Unsupervised clustering identified CIMP subtypes. Integrative analyzes included promoter-focused methylation–expression coupling (ELMER), immune deconvolution (GSVA, MCPcounter, ESTIMATE), genomic profiling, and drug sensitivity prediction (GDSC). Clinical relevance was further assessed in an independent ICB-treated cohort (PRJEB23709). Functional assays evaluated the role of NRAS in melanoma cell lines.

**Results:**

Two epigenetic subtypes were defined: CIMP+ (24.9%) and CIMP− (75.1%). CIMP+ tumors were associated with older age and male sex and exhibited significantly worse overall and progression-free survival, remaining an independent prognostic factor after multivariable adjustment. Integrative analysis identified 2,510 hypermethylated promoter probes linked to reduced expression of 1,707 genes enriched in interferon-γ and inflammatory pathways, although these associations likely reflect both tumor-intrinsic regulation and differences in cellular composition. Consistently, CIMP+ melanomas displayed an immune-depleted bulk-tumor profile, with reduced immune checkpoint gene expression, lower immune/stromal scores, and decreased leukocyte-associated methylation signals. Genomically, CIMP+ tumors were enriched for NRAS and ARID2 mutations, showed fewer BRAF alterations, and exhibited significantly increased copy-number alterations and chromosomal instability. In an ICB-treated cohort, transcriptome-inferred CIMP+ status was significantly enriched in non-responders and associated with inferior survival. Drug sensitivity modeling identified multiple candidate compounds, including elesclomol and Wnt pathway inhibitors. Functionally, NRAS knockdown suppressed proliferation, migration, and invasion while promoting apoptosis and epithelial–mesenchymal transition reversal.

**Conclusion:**

CIMP defines a clinically relevant melanoma subtype characterized by epigenetic remodeling, genomic instability, and an immune-poor tumor microenvironment, and is associated with poor prognosis and reduced benefit from immunotherapy. These findings suggest that therapeutic resistance in CIMP+ melanoma is multifactorial and highlight potential avenues for targeted intervention.

## Introduction

Cutaneous melanoma (CM) is among the most lethal skin cancers, with an increasing incidence worldwide (1). Although early-stage melanoma is often curable with surgical excision, patients with advanced disease face dramatically worse outcomes (2). Under AJCC8 staging, stage I patients have a 5-year survival rate of 97–99%, while stage IV metastatic melanoma patients have a survival rate of approximately 30% (3). Survival rates for stage II–III melanoma vary widely (32–94%) due to biologically distinct subtypes with differing levels of aggressiveness, metastatic potential, and immune evasion (4). This clinical heterogeneity underscores the need for robust molecular biomarkers to complement traditional staging and guide therapeutic decisions.

Immune checkpoint blockade (ICB) has revolutionized melanoma therapy. Antibodies targeting PD-1, CTLA-4, and LAG-3 have demonstrated durable responses in subsets of patients, but only around 10% of metastatic melanoma patients respond to CTLA-4 based therapy, and many eventually acquire resistance (5, 6). The presence of tumor-infiltrating lymphocytes, especially CD8^+^ T cells, is linked to better survival rates, whereas tumor-associated macrophages and immunosuppressive stromal cells are related to unfavorable outcomes (7). Scoring systems such as ISTMEscore and TMEscore have been developed to quantify immune infiltration and predict prognosis, yet they often focus on individual cell types and fail to capture complex interactions within the tumor microenvironment (TME) (8, 9).

DNA methylation represents a stable and clinically translatable layer of epigenetic regulation that influences gene expression, immune evasion, and tumor progression (10). Aberrant methylation at CpG dinucleotides can silence genes critical for antigen presentation, interferon signaling, and apoptosis (11, 12). Unlike transcriptomic signatures, methylation patterns are chemically stable, reproducible across platforms, and assayable in both fresh-frozen and archival tissues, making them ideal candidates for biomarker development. Previous studies have revealed that promoter hypermethylation of tumor suppressor genes, as well as hypomethylation of gene bodies, can drive melanoma progression (13). Notably, UV irradiation can remodel methylation patterns: UVA-exposed melanoma cells co-cultured with senescent fibroblasts show hypomethylation of the GDF-15 promoter and elevated paracrine IL-6 signaling, promoting survival under genotoxic stress (14).

A key aspect of melanoma epigenetics is the CpG island methylator phenotype (CIMP), marked by extensive hypermethylation in CpG-rich areas (15). In melanoma, CIMP has been associated with advanced tumor stage, poor survival, and reduced immune infiltration (16). However, most studies have focused on late-stage primary or metastatic tumors, and the prevalence and biological relevance of CIMP in early-stage melanomas remain unclear. Moreover, while promoter regions have been the primary focus, non-promoter regions such as gene bodies and intergenic elements also play important regulatory roles in gene expression and tumor progression, yet remain understudied.

Given the interplay between DNA methylation and immune function, integrating epigenetic and immune data holds promise for identifying predictive biomarkers of immunotherapy response. This study conducted a genome-wide analysis of DNA methylation and transcriptomic profiles in primary melanomas to identify methylation-defined subtypes linked to immune context and clinical outcomes. We demonstrate that a subset of melanomas exhibits CpG island hypermethylation accompanied by transcriptional repression of immune-related pathways and an immune-depleted microenvironment, correlating with worse survival and resistance to checkpoint blockade. Our results highlight DNA methylation as a stable, clinically actionable biomarker to refine risk stratification and guide immunotherapy in melanoma.

## Materials and methods

### Multi-omics data sets of skin cutaneous melanoma from TCGA

Multi-omics data of skin cutaneous melanoma (SKCM) were obtained from The Cancer Genome Atlas (TCGA) (17). DNA methylation profiles, quantified using the Illumina HumanMethylation450 BeadChip platform, were downloaded from the UCSC Xena browser (https://xenabrowser.net/) under the TCGA-SKCM project, including 455 primary or metastatic melanoma samples and two adjacent normal tissues. Gene expression data were retrieved for 453 primary or metastatic melanoma samples (18) and were originally quantified as fragments per kilobase of transcript per million mapped reads (FPKM). FPKM values were subsequently converted to transcripts per kilobase million (TPM) to improve comparability across samples and consistency with microarray-based analyzes. Notably, all tumors with transcriptomic data also had matched DNA methylation profiles. Copy number variation (CNV) segment data were obtained from FireBrowse (http://firebrowse.org/). Somatic mutation data, clinicopathological characteristics, and clinical outcomes, including progression-free survival (PFS) and overall survival (OS), were collected from cBioPortal (https://www.cbioportal.org/).

### External epigenetic and transcriptomic datasets of cutaneous melanoma

Given the limited availability of normal samples in the TCGA cohort, genome-wide DNA methylation data from the Conway cohort (GSE120878) were incorporated (19). This dataset comprised 89 primary invasive FFPE melanoma samples and 73 benign nevi, profiled using the Illumina Infinium HumanMethylation450 BeadChip platform. Benign nevi were incorporated as lineage-matched, non-malignant melanocytic comparators to expand the reference baseline under the constraint of very limited adjacent normal tissues in TCGA. However, because nevi are biologically distinct from normal skin and represent a surrogate rather than an ideal normal control, their use was intended to provide a pragmatic low-methylation reference state for probe selection and external contextualization, not a definitive normal-versus-tumor contrast.

An independent transcriptomic cohort was obtained from a published clinical dataset (PRJEB23709; Gide cohort), which included 91 patients with metastatic melanoma treated with immune checkpoint blockade. Patients received either anti–PD-1 monotherapy (nivolumab or pembrolizumab; n = 50) or combined anti–CTLA-4 plus anti–PD-1 therapy (ipilimumab with nivolumab or pembrolizumab; n = 41). Within this cohort, 57 patients were classified as responders and 34 as non-responders. Corresponding PFS and OS data were extracted from the original publication (20).

### DNA methylation data preprocessing and batch correction

To address the scarcity of adjacent normal tissues in the TCGA cohort, methylation data from TCGA-SKCM and the Conway cohort were merged, yielding a combined dataset of 615 samples derived from two independent batches. Potential batch effects were corrected using the ComBat algorithm implemented in the R package sva (21), which applies an empirical Bayes framework to adjust for technical variation. Because direct application of ComBat to β values may generate values outside the [0,1] range, β values were first transformed to M values using a logit transformation, followed by ComBat correction and inverse logit transformation, as implemented in the ChAMP pipeline. Comprehensive probe filtering was subsequently performed using ChAMP. Specifically, we excluded 1,085 non-CpG probes, 20,849 probes containing single-nucleotide polymorphisms (SNPs) at or near the targeted CpG site, four probes mapping to multiple genomic locations, and 7,515 probes located on the X or Y chromosomes (22, 23). After filtering, 318,826 probes across 615 samples were retained for downstream analyzes.

### Identification of the CIMP

To define CpG island methylator phenotypes, we first selected probes exhibiting low methylation levels in normal tissues (mean β value < 0.2 across 75 normal samples, including nevi and adjacent normal tissues) and high variability in tumors (standard deviation > 0.2). This filtering scheme was designed to enrich for CpGs that acquire aberrant methylation in melanoma while retaining intertumoral discriminatory power; nevertheless, because it preferentially captures loci with low baseline methylation, it may favor the identification of hypermethylated CpGs and should therefore be viewed as an enrichment strategy rather than as direct proof of a discrete methylator class. Unsupervised hierarchical clustering was performed on the β values of these probes in the 453 TCGA tumor samples using Euclidean distance and Ward’s linkage method. The resulting dendrogram was cut at k = 2 to define two methylation-based subtypes, because the dominant split separated tumors with globally high versus low methylation and most closely matched the biologically interpretable CIMP-like axis. We nevertheless treated cluster number as a modeling choice and used the external cohort primarily to examine transferability of this high-/low-methylation structure and its biological correlates, rather than to claim *de novo* rediscovery of an identical class architecture. Although β values were used for clustering, M values—owing to their superior statistical properties—were additionally used for data visualization (24).

Rationale for probe thresholds and clustering parameters. The beta-value threshold of <0.2 in non-malignant reference samples was selected to focus on CpG loci with low baseline methylation and sufficient dynamic range for detecting tumor-associated methylation gain, whereas the tumor SD > 0.2 criterion was used to retain probes with substantial intertumoral variability and to avoid clustering driven by largely invariant loci. These thresholds were used as pragmatic enrichment filters rather than biologically absolute boundaries. The primary clustering was performed using the filtered beta-value matrix, Euclidean distance, and Ward linkage, with k = 2 selected to capture the dominant high- versus low-methylation axis that most closely corresponded to a biologically interpretable CIMP-like pattern. Because different clustering resolutions may reveal additional lower-level heterogeneity, the binary CIMP+/CIMP− assignment was treated as a major methylation-stratification axis rather than an exhaustive epigenetic taxonomy. Robustness was further evaluated by examining transferability of the same methylation axis in the external Conway cohort and by assessing whether the CIMP-associated transcriptomic program could be projected into an independent ICB-treated cohort; however, comprehensive bootstrap resampling, consensus clustering, and systematic benchmarking across all alternative clustering algorithms were not performed.

### Estimation of tumor microenvironment cell abundance

Tumor microenvironment cell infiltration was estimated using previously described gene signatures derived from MCPcounter and CIBERSORT (25–27). A total of 364 genes representing 24 immune and stromal cell populations were compiled. Gene set variation analysis (GSVA) was conducted using the R package GSVA to generate enrichment scores for each cell type. The overall infiltration of immune and stromal components was further quantified using the ESTIMATE algorithm (28). Because these approaches infer relative signatures from bulk expression profiles, the resulting scores were interpreted primarily as composite measures of immune/stromal admixture that may also be influenced by tumor purity, rather than as direct quantification of specific immune populations. Formal purity-adjusted regression analyzes were not performed in the present study. In addition, tumor-infiltrating lymphocyte methylation (MeTIL) scores were calculated for samples from the TCGA and Conway cohorts according to a previously published framework (29).

### Differential analyzes and functional enrichment

Differentially methylated probes (DMPs) were identified using ChAMP. Probes were considered significantly hypermethylated if the mean β value exceeded 0.5 in one subtype and was below 0.3 in the reference group, with P < 0.05 and false discovery rate (FDR) < 0.05; hypomethylated probes were defined using inverse criteria. Functional enrichment analysis at the CpG level was performed using the missMethyl package, applying a generalized gene set test (GGST) with Hallmark gene sets from the Molecular Signatures Database (MSigDB) (30, 31) as background. Differential gene expression analysis was conducted using limma (32). For gene set enrichment analysis (GSEA), pre-ranked gene lists were generated and analyzed with clusterProfiler against Hallmark pathways (33). Gene list–based functional enrichment analyzes were additionally performed using Enrichr (34).

### Analysis of methylation–expression coupling

To investigate putative methylation-associated transcriptional repression, integrative analyzes of DNA methylation and gene expression were performed using the R package ELMER (35). Although ELMER is commonly applied to infer distal enhancer–gene regulatory relationships, we restricted the present analysis to promoter-associated probes based on Infinium HumanMethylation450K annotations because our primary objective was to determine whether the CIMP-defining hypermethylated state was accompanied by coordinated methylation–expression coupling at proximal regulatory regions. This promoter-focused application was therefore used as a conservative association screen rather than as a canonical enhancer-target discovery workflow. Candidate genes were defined as those significantly downregulated in association with promoter hypermethylation (Δβ ≥ 0.2, FDR < 0.05).

For each candidate probe, the 20 nearest upstream and downstream genes were examined. Associations between probe methylation and gene expression were assessed using the Mann–Whitney U test, testing the null hypothesis that gene expression in the target group was less than or equal to that in the reference group. The resulting methylation–expression pairs were interpreted as evidence of association and putative repression, rather than as direct proof of causal epigenetic silencing.

### Prediction of therapeutic response

For chemotherapy sensitivity prediction, drug response and phenotypic data were obtained from the Genomics of Drug Sensitivity in Cancer (GDSC) 2016 dataset. The R package pRRophetic was applied to predict drug sensitivity for each melanoma sample, using gene expression profiles from 727 human cancer cell lines as the training set (36). Ridge regression was employed to estimate half-maximal inhibitory concentration (IC50) values, and predictive performance was evaluated using 10-fold cross-validation.

For immunotherapy response prediction, a published transcriptomic dataset comprising 47 melanoma patients responsive to immune checkpoint blockade was retrieved (37). Subclass mapping was applied to infer clinical response to immune checkpoint inhibitors by comparing transcriptional similarity between datasets (38).

Transcriptome-based inference of CIMP status in the ICB-treated cohort was performed as a surrogate transfer analysis because DNA methylation profiles were not available for these patients. Briefly, genes differentially expressed between methylation-defined CIMP+ and CIMP− tumors in the TCGA discovery cohort were ranked by subtype specificity, and the top 100 genes preferentially upregulated in each subtype were combined to generate a 200-gene nearest-template prediction (NTP) signature. After restricting the analysis to genes shared between the TCGA and Gide expression matrices and applying expression-scale normalization, each Gide sample was assigned to the closest CIMP-associated transcriptomic template. To provide an orthogonal check, we also performed supervised hierarchical clustering using the 1,707 genes showing promoter hypermethylation-associated reduced expression in TCGA and compared the resulting grouping with the NTP-based labels. Because these labels were inferred from transcriptomic data, they were interpreted as CIMP-associated expression states rather than methylation-confirmed CIMP classes.

### Characterization of molecular and clinical subtype features

Comprehensive characterization of epigenetic subtypes was conducted using the R package MOVICS with default parameters (39). Comparisons between subtypes included somatic mutation frequencies, fraction of genome altered (FGA), and clinical characteristics. Mutation landscapes and putative driver mutations were analyzed using maftools, based on previously reported melanoma driver genes (40, 41). Recurrent focal somatic copy number alterations were identified using GISTIC2.0 implemented through GenePattern (42). Thresholds for amplification and deletion were set at 0.2, with a q value cutoff of 0.05 and a confidence level of 95%.

### Human tissue specimens

Fresh tumor tissues and matched adjacent non-tumor tissues were collected from five patients who underwent surgical resection for primary cutaneous melanoma. Normal tissues were obtained at least 2 cm away from the tumor margin and were histologically confirmed to be tumor-free. The study was approved by the Institutional Review Board of the participating hospital, and written informed consent was obtained from all patients. Immediately after surgical excision, tissues were rinsed with ice-cold phosphate-buffered saline (PBS), snap-frozen in liquid nitrogen within 10 min, and stored at −80 °C until RNA isolation.

### Cell culture

Human melanoma cell lines TE353.sk, SK-MEL-31, COLO 829, G-361, A375, and SK-MEL-28 were obtained from the American Type Culture Collection (ATCC, USA). Cells were authenticated by short tandem repeat profiling and routinely tested for mycoplasma contamination. TE353.sk, SK-MEL-31, COLO 829, G-361, and A375 cells were maintained in high-glucose Dulbecco’s modified Eagle’s medium (DMEM; HyClone, Cat# SH30022.01), whereas SK-MEL-28 cells were cultured in McCoy’s 5A medium (Gibco, Cat# 16600082). Media were supplemented with 10% fetal bovine serum (FBS; HyClone, Cat# SH30084.03) and 1% penicillin–streptomycin solution (KeyGEN, Cat# KG1105). Cells were incubated at 37 °C in a humidified atmosphere containing 5% CO_2_ and passaged using 0.25% trypsin–EDTA (Gibco, Cat# 25200056). Experiments were performed using cells in logarithmic growth phase within 10 passages.

### siRNA transfection

NRAS-targeting small interfering RNAs and a non-targeting negative control (si-NC) were synthesized by Sangon Biotech (Shanghai, China). Cells were seeded in six-well plates and transfected at approximately 70–80% confluence using Lipofectamine 3000 reagent (Thermo Fisher Scientific, Cat# L3000015) according to the manufacturer’s instructions. Briefly, siRNA and Lipofectamine were diluted separately in Opti-MEM reduced-serum medium (Gibco, Cat# 31985070), combined for 20 min at room temperature to allow complex formation, and added to the cells to achieve a final siRNA concentration of 50 nM. After 6 h, the medium was replaced with fresh complete medium. Cells were harvested 24–48 h post-transfection for downstream analyzes. Knockdown efficiency was confirmed by qRT-PCR in each independent experiment.

### RNA extraction and quantitative real-time PCR

Total RNA was extracted using TRIzol reagent (Takara, Cat# 9109) following the manufacturer’s protocol. RNA purity and concentration were measured using a NanoDrop spectrophotometer. Samples with A260/A280 ratios between 1.8 and 2.0 were used for subsequent analysis. Genomic DNA contamination was removed and reverse transcription was performed using the PrimeScript RT reagent kit (Takara, Cat# RR047A). Quantitative PCR was conducted using SYBR GreenER Supermix (Takara, Cat# RR820A) on a LightCycler 480 system (Roche, Switzerland). Relative expression was calculated using the 2^-^ΔΔCt method with β-actin as an internal control. All reactions were performed in triplicate, and at least three independent biological replicates were included.

### Cell proliferation assay

Cell proliferation was assessed using a Cell Counting Kit-8 (CCK-8; Beyotime, C0037). Twenty-four hours after transfection, cells were seeded into 96-well plates at a density of 4 × 10³ cells per well. At the indicated time points, 10 μL of CCK-8 solution was added to each well and incubated for 1.5 h at 37 °C. Absorbance at 450 nm was measured using a microplate reader (BioTek). Each condition was tested in triplicate wells, and experiments were repeated independently three times.

### Apoptosis analysis

Apoptosis was analyzed using an Annexin V-FITC/PI apoptosis detection kit (Beyotime, C1062S) according to the manufacturer’s instructions. After transfection, cells were harvested, washed with cold PBS, and resuspended in binding buffer followed by incubation with Annexin V-FITC and propidium iodide for 15 min in the dark at room temperature. Samples were analyzed immediately on a BD FACSCalibur flow cytometer (BD Biosciences), and data were processed using FlowJo software (Tree Star). Early and late apoptotic cells were combined for quantification. Three independent experiments were performed.

### Migration and invasion assays

Cell migration and invasion were evaluated using Transwell chambers with 8-μm pore size inserts (Corning, Cat# 3422). For invasion assays, inserts were pre-coated with Matrigel (BD Biosciences, Cat# 356234). Transfected cells suspended in serum-free medium were added to the upper chamber, while the lower chamber contained medium supplemented with 10% FBS as a chemoattractant. After 24 h incubation, cells on the upper surface were removed, and migrated or invaded cells were fixed with 4% paraformaldehyde, stained with 0.1% crystal violet, and counted under a light microscope in five randomly selected fields. Each experiment was performed in triplicate.

### Western blot analysis

Cells were lysed using RIPA buffer (Beyotime, Cat# P0013B) supplemented with protease and phosphatase inhibitors (Beyotime, Cat# P1045). Protein concentrations were determined using a BCA protein assay kit (Yamei Bio, Cat# ZJ101). Equal amounts of protein (30–50 μg) were separated on 10% SDS-PAGE gels and transferred to PVDF membranes (Millipore, Cat# IPVH00010). Membranes were blocked with 5% non-fat milk in TBST and incubated overnight at 4 °C with primary antibodies against cleaved caspase-3 (CST, Cat# 9661, 1:1000), Bcl-2 (Abcam, Cat# ab182858, 1:2000), E-cadherin (Abcam, Cat# ab1416, 1:1000), Vimentin (Abcam, Cat# ab92547, 1:1000), and β-actin (Abcam, Cat# ab179511, 1:1000). After incubation with HRP-conjugated secondary antibody (Abcam, Cat# ab205718, 1:2000), signals were visualized using enhanced chemiluminescence (Yamei Bio, Cat# SQ201) and quantified using ImageJ software. Experiments were independently repeated three times.

### Statistical analyzes

All statistical analyzes were performed using R version 4.0.2. Fisher’s exact test was applied to categorical variables, while two-sided Mann–Whitney U tests were used for continuous variables. Survival differences were assessed using Kaplan–Meier analysis with log-rank tests, and hazard ratios (HRs) with 95% confidence intervals (CIs) were estimated using Cox proportional hazards regression models. For all analyzes, a two-sided P value < 0.05 was considered statistically significant.

## Results

### CIMP in skin cutaneous melanoma is associated with unfavorable clinical outcomes

**Figure 1:**
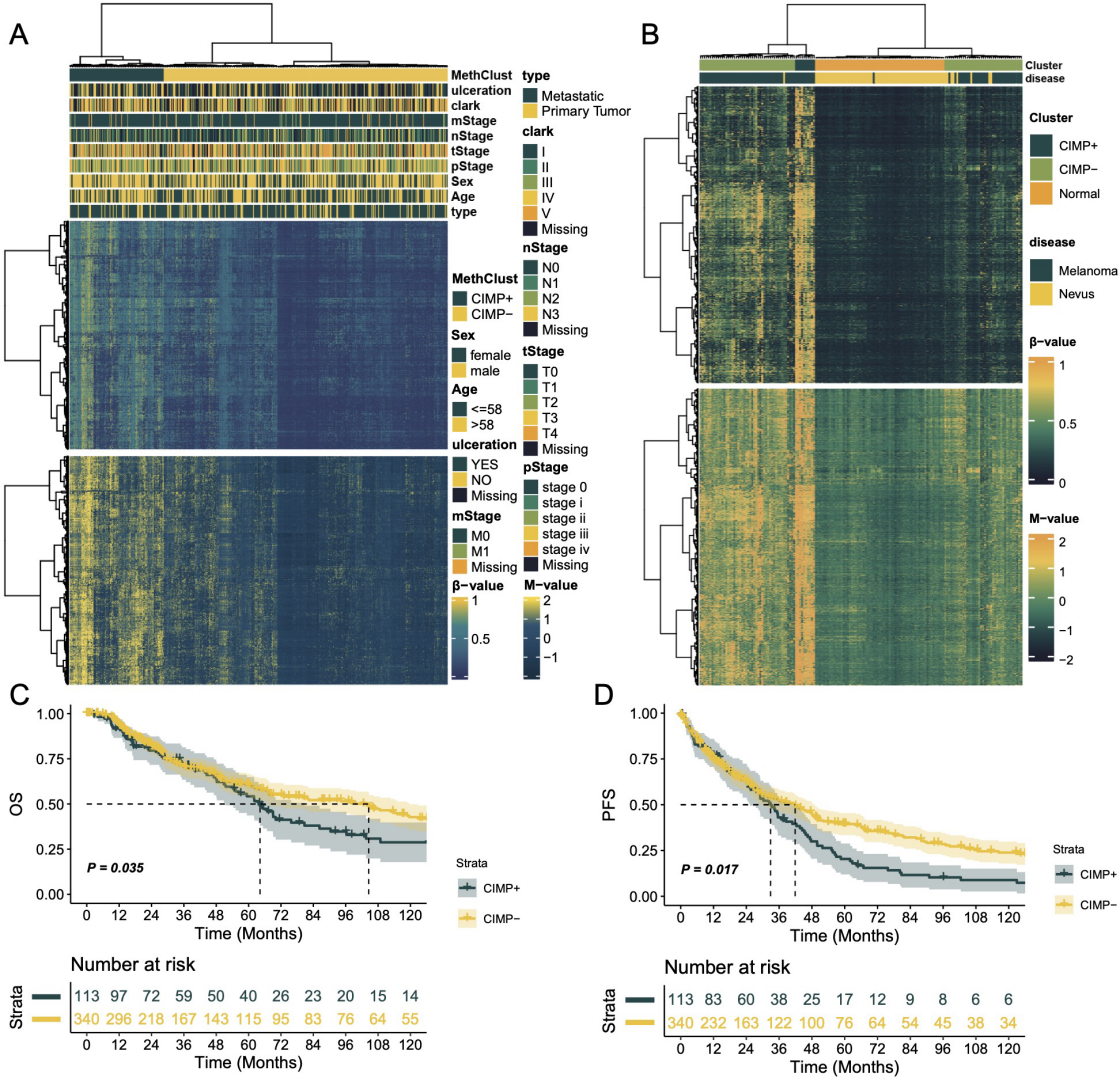
DNA methylation landscape and epigenetic subtypes in cutaneous melanoma. **(A)** Unsupervised hierarchical clustering of 453 TCGA-SKCM tumors using 7,738 variably methylated CpG probes identified CIMP+ and CIMP− subtypes. Upper and lower heatmaps display β-values and M-values, respectively. β-values represent methylation proportions (0–1), whereas M-values are logit-transformed methylation values. **(B)** Supervised clustering of the external Conway cohort using the same probe set identified in TCGA. Nevi samples were included as non-malignant melanocytic reference samples. **(C, D)** Kaplan–Meier analyzes of overall survival (OS) **(C)** and progression-free survival (PFS) **(D)** in the TCGA cohort according to CIMP status.

We examined DNA methylation profiles from 453 primary or metastatic cutaneous melanoma cases in the TCGA cohort using the Illumina HumanMethylation450K platform. We analyzed 73 nevi samples from Conway’s cohort alongside two adjacent normal tissues from TCGA to identify probes showing low baseline methylation in lineage-relevant non-malignant reference tissues. Because this reference set combined benign nevi with the very limited adjacent tissues available in TCGA, the filtering strategy was intended to enrich for CpGs gaining methylation in melanoma, but it may also preferentially retain hypermethylated loci. We further refined the probe set by selecting those with high variability (SD > 0.2) in the 453 tumor samples, resulting in a final set of 7,738 probes. Unsupervised hierarchical clustering of these probes revealed a major split between a smaller group (n = 113, 24.9%) with significantly higher DNA methylation levels, labeled CIMP+, and a larger group (n = 340, 75.1%) with low DNA methylation, labeled CIMP− (**Figure 1A**). To assess the robustness of this methylation pattern in cutaneous melanoma, we performed supervised clustering on Conway’s cohort using the same 7,738 probes, which generated three clusters. This configuration likely reflected separation of non-malignant nevi from melanoma together with residual heterogeneity among melanoma samples; importantly, among melanoma cases, a hypermethylated subgroup analogous to CIMP+ remained evident (**Figure 1B**). Accordingly, the external analysis supports transferability of the dominant high-/low-methylation axis, although it does not by itself establish complete invariance of cluster structure across cohorts. We further explored the clinicopathological associations of these subgroups. CIMP+ was significantly correlated with older age (P = 0.006) and male sex (P = 0.013). CIMP+ tumors also showed trends toward more advanced stages (P = 0.094) and increased mitotic counts (P = 0.096), but these associations did not reach statistical significance and should therefore be interpreted cautiously. Importantly, these epigenetic subgroups were independent of tumor type (primary vs. metastatic, P = 0.6) (**Supplementary Table 1**). Survival analysis indicated that patients with CIMP+ melanoma exhibited significantly worse overall survival (OS; P = 0.035) and progression-free survival (PFS; P = 0.017) than CIMP− patients, underscoring the prognostic relevance of this methylation-defined stratification (**Figures 1C, D**).

To improve reproducibility of the classification, the above thresholds and clustering settings are now explicitly reported. We emphasize that the SD and beta-value cutoffs were used to enrich for variable CpGs with low non-malignant baseline methylation, and that k = 2 was chosen to summarize the dominant methylation split rather than to exclude the possibility of additional epigenetic substructure. Thus, the survival and biological comparisons should be interpreted as associations with this predefined CIMP-associated methylation axis.

### CIMP+ melanomas show promoter hypermethylation associated with reduced expression of immune-related genes

**Figure 2:**
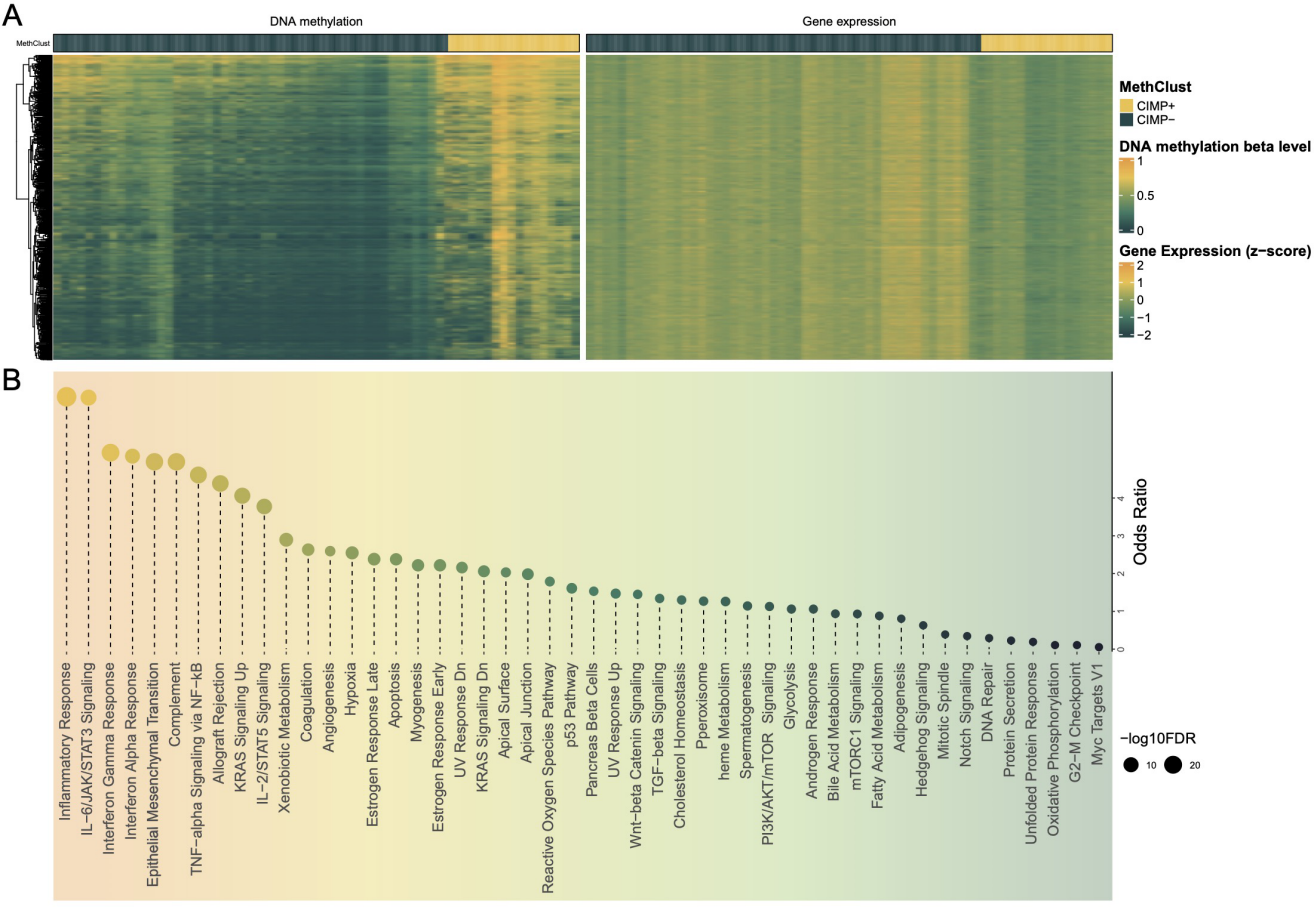
Promoter methylation–expression coupling in immune-related genes of CIMP+ melanoma. **(A)** Heatmap showing promoter-associated DNA methylation and gene expression profiles of 8,448 promoter-gene pairs in CIMP+ and CIMP− melanoma. **(B)** Lollipop plot displaying enrichment results for genes associated with promoter hypermethylation and reduced expression in CIMP+ versus CIMP− melanoma.

We applied the promoter-focused ELMER framework to integrate DNA methylation and transcriptomic data. Because CIMP status was itself derived from methylation patterns, the enrichment of hypermethylated promoter probes in CIMP+ is directionally concordant with the subtype definition and should therefore not be considered an independent validation of the class. Within this association-based framework, we identified 2,510 promoter probes with a differential β-value > 0.2 (FDR < 0.05) between CIMP+ and CIMP−, corresponding to 8,448 promoter-gene pairs involving 1,707 genes (**Figure 2A**; **Supplementary Table 2**–**S4**). Genes linked to these promoter-associated methylation changes were significantly enriched for inflammatory response and interferon-γ signaling pathways (**Figure 2B**; **Supplementary Table 5**). Given the immune-depleted phenotype of CIMP+ melanomas, however, these transcriptomic differences likely reflect both methylation-associated repression within tumor cells and reduced abundance of immune cell populations in the bulk samples, and thus should not be interpreted as proof of purely tumor-intrinsic epigenetic silencing.

Mechanistically, this pattern suggests a regulatory cascade in which CpG island hypermethylation may weaken tumor-intrinsic inflammatory competence before overt immune-cell depletion becomes apparent. In particular, coordinated promoter methylation coupled with lower expression of interferon-γ response and antigen-processing programs could reduce melanoma-cell responsiveness to immune stimulation, impair chemokine-mediated T-cell recruitment, and decrease the availability of antigen-presentation cues required for productive cytotoxic T-cell recognition. Although this analysis remains associative, the pathway-level concordance provides a biologically plausible link between the CIMP epigenetic state and the immune-poor phenotype observed in bulk tumors.

### CIMP+ melanomas are associated with an immune-depleted bulk-tumor profile

**Figure 3:**
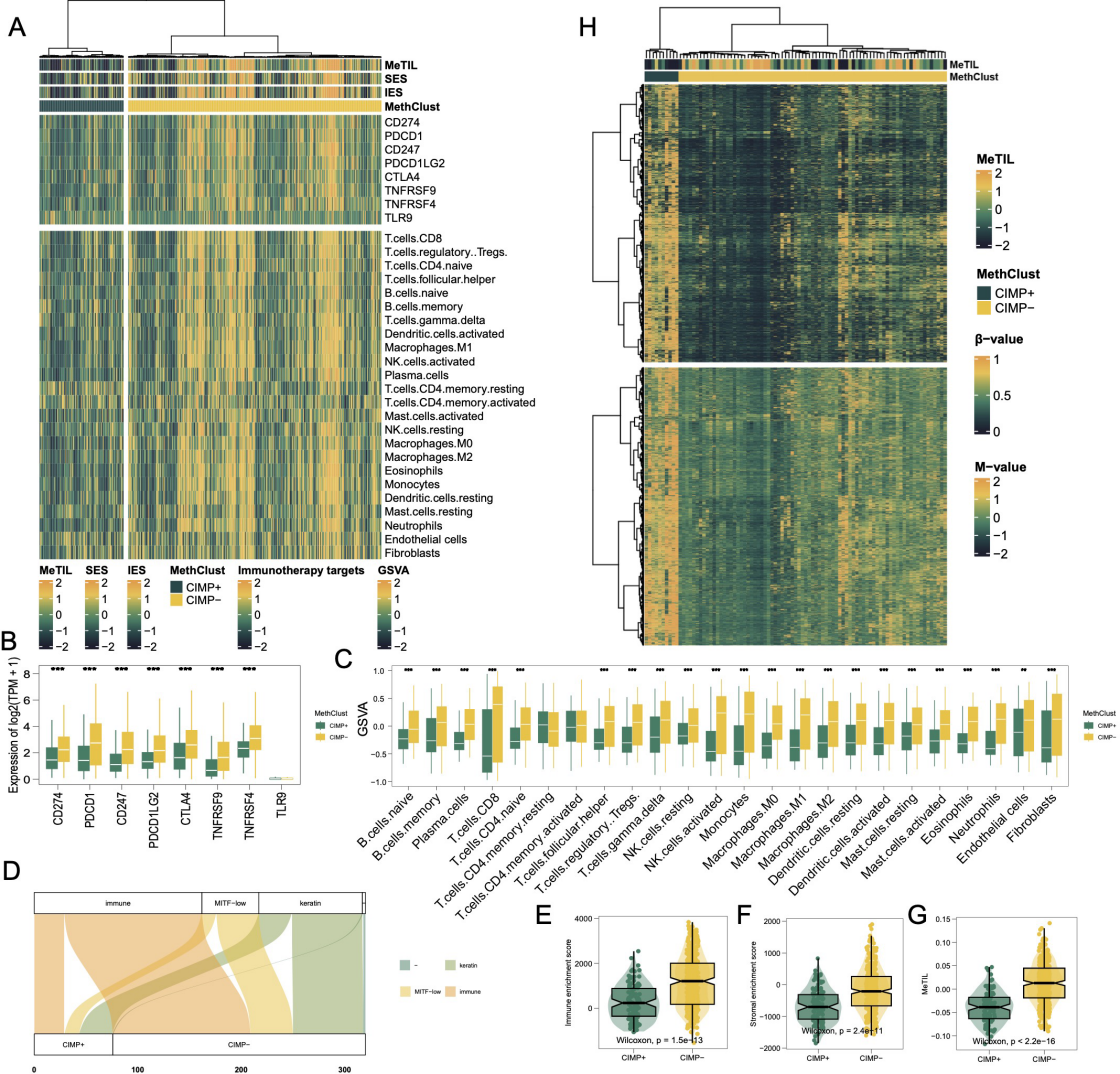
Tumor microenvironment characteristics of CIMP+ melanoma. **(A)** Heatmap showing differential immune checkpoint expression, immune cell infiltration and stromal cell infiltration between CIMP+ and CIMP- melanoma subgroups, based on DNA methylation data. **(B)** Boxplots displaying the differential expression of immune checkpoint genes between CIMP+ and CIMP- melanoma subgroups. **(C)** Boxplots representing GSVA enrichment scores for immune and stromal cell infiltration in CIMP+ vs. CIMP- melanoma. **(D)** Sankey diagram illustrating the distribution of immune, MITF-low and keratin subtypes between CIMP+ and CIMP- melanoma subgroups. **(E)** Boxplot showing immune scores in CIMP+ vs. CIMP- melanoma. **(F)** Boxplot depicting stromal scores in CIMP+ vs. CIMP- melanoma. **(G)** Boxplot of MeTIL scores in CIMP+ vs. CIMP- melanoma. **(H)** Validation of immune-depleted phenotype in Conway’s cohort, with significantly lower MeTIL score in CIMP+ compared to CIMP- (P = 0.001). Statistical significance: ***P < 0.001.

We next examined whether CIMP status was associated with bulk-tumor immune and stromal signatures within the tumor microenvironment (TME) (**Figure 3A**). CIMP+ tumors showed significantly lower transcript levels of immune checkpoint-related genes, including PDCD1 (PD1), CD247 (CD3), CD274 (PDL1), PDCD1LG2 (PDL2), CTLA4, TNFRSF9 (CD137), and TLR9 (**Figure 3B**). Because these transcripts in bulk melanoma samples are strongly influenced by infiltrating immune cells, this pattern is more appropriately interpreted as reflecting reduced immune-cell admixture and altered tumor composition than definitive tumor-intrinsic downregulation. Consistently, GSVA-based signatures indicated lower immune and stromal enrichment scores in CIMP+ melanomas for 22 of 24 cell populations (**Figure 3C**). However, because these estimates were derived from bulk transcriptomic data, they primarily capture relative differences in overall immune/stromal content, with possible contribution from tumor purity, rather than unequivocal alterations in each specific immune-cell subset. In line with this interpretation, only 17.9% of tumors assigned to the previously reported RNA-seq-defined “immune” subtype were CIMP+ (**Figure 3D**), and ESTIMATE likewise showed significantly lower immune and stromal scores in CIMP+ tumors (**Figures 3E, F**). The MeTIL score, which reflects leukocyte-associated methylation patterns, was also reduced in CIMP+ melanomas (**Figure 3G**). Unsupervised clustering of 89 melanoma cases from Conway’s cohort confirmed the immune-depleted phenotype, showing that CIMP+ tumors had significantly lower MeTIL scores than CIMP- tumors (P = 0.001; **Figure 3H**). Together, these results support an immune-poor bulk-tumor profile in CIMP+ melanoma, although formal purity-adjusted modeling was not performed and cell-type-specific inferences should therefore be made cautiously. Importantly, these findings do not exclude other contributors to immune depletion, including chromosomal instability, antigen-presentation defects, stromal remodeling, and oncogenic signaling programs that may operate alongside CIMP-associated methylation changes.

### Genomic heterogeneity and chromosomal instability in CIMP+ melanomas

**Figure 4:**
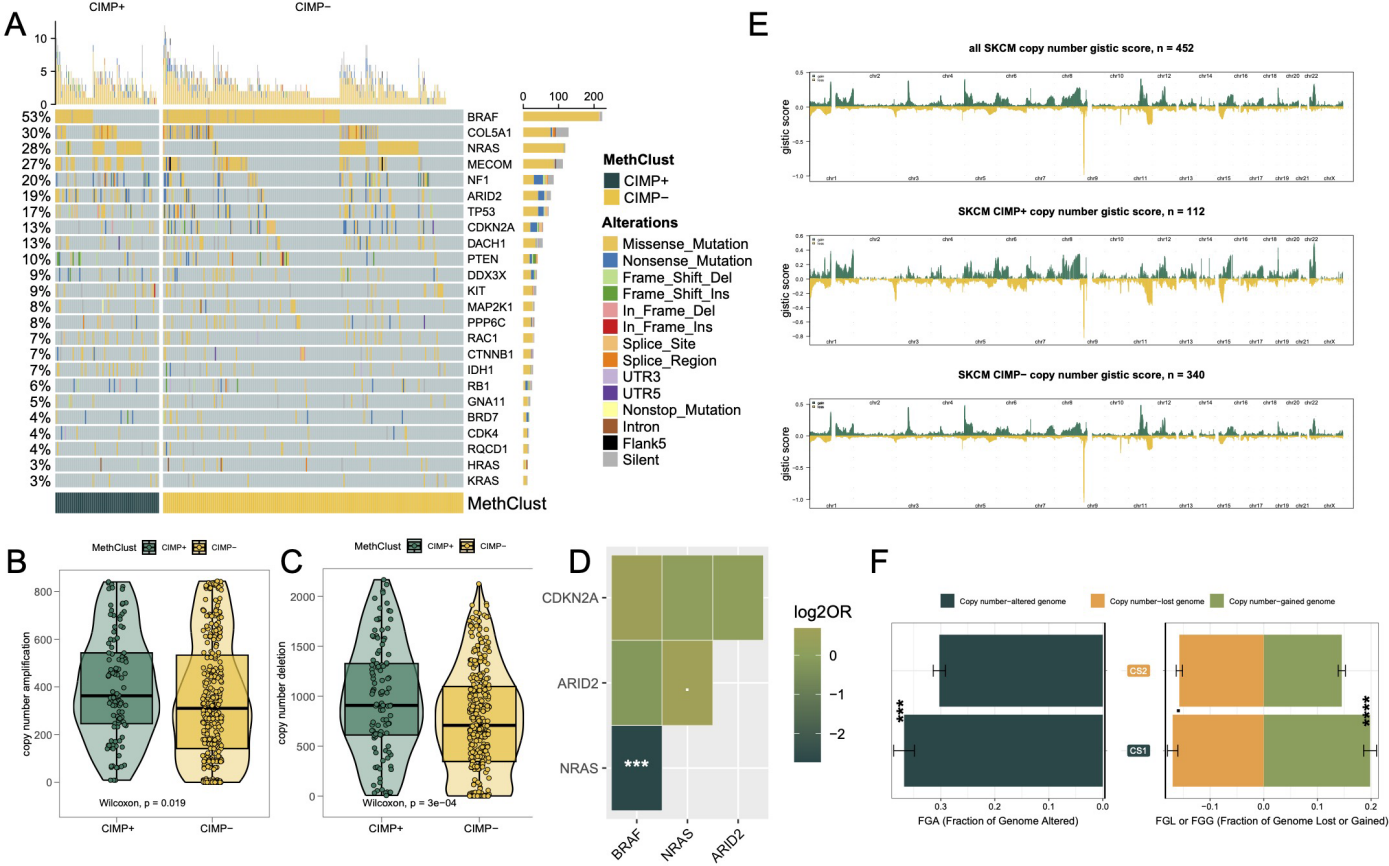
Genomic heterogeneity and chromosomal instability in CIMP+ melanoma. **(A)** Differential mutational landscape between CIMP+ and CIMP- subgroups. **(B, C)** Copy number analysis revealed that CIMP+ melanoma presented with significantly more focal-level amplifications **(B)** and deletions **(C)** compared to CIMP-. **(D)** Heatmap illustrating the correlation between mutations in BRAF, NRAS, ARID2, and CDKN2A. **(E)** GISTIC score peak plots representing copy number variations (CNVs) in cutaneous melanoma. The top plot shows the overall CNV profile across 452 SKCM samples. The middle plot represents CNVs in the CIMP+ subgroup (n = 112), and the bottom plot displays CNVs in the CIMP- subgroup (n = 340). **(F)** Bar plots showing the distribution of copy number-altered genomes, including copy number losses and gains, in CIMP+ vs. CIMP- melanoma. CS1 and CS2 correspond to CIMP+ and CIMP− subtypes, respectively.

To investigate the genomic heterogeneity of cutaneous melanoma, we analyzed mutational landscape and identified 80 genes that showed differential mutational frequency between two phenotypes (P < 0.05) with overall mutational rate greater than 10% across the entire cohort (**Figure 4A**). Within those genes that were previously identified as driver mutations for cutaneous melanoma (40), we found that CIMP+ harbored significantly more ARID2 (32 [29.6%] vs 38 [12.1%; P < 0.001]) and NRAS (44 [40.7%] vs 75 [24%%; P < 0.001]) mutations as compared with CIMP-; while CIMP- presented with more mutations of BRAF (182 [58.1%] vs 39 [36.1%; P < 0.001]) and CDKN2A (48 [15.3%] vs 6 [5.6%; P < 0.001]) mutations (**Supplementary Table 6**). Analysis revealed that CIMP+ cutaneous melanomas exhibited significantly higher focal-level copy number amplifications (**Figure 4B**) and deletions (**Figure 4C**) compared to CIMP- melanomas (both, P < 0.001). Of note, NRAS showed significant mutual exclusivity concerning BRAF mutation (P < 0.001) while tending to present with a co-occurrence pattern with ARID2 (P = 0.09) (**Figure 4D**, **Supplementary Table 7**). We analyzed the broad-level CNA across the entire cohort and within two epigenetic subgroups. The focal CNA landscape in CIMP+ was notably more unstable compared to CIMP- (**Figure 4E**). We assessed chromosomal instability using FGA scores and found that CIMP+ exhibited greater chromosomal instability compared to CIMP-, as evidenced by significantly higher copy number variations (P < 0.001; **Figure 4F**).

The enrichment of NRAS and ARID2 alterations in CIMP+ tumors further supports a genetic-epigenetic interaction model. ARID2 loss may compromise PBAF-mediated chromatin accessibility and thereby favor abnormal promoter methylation or transcriptional repression, whereas oncogenic NRAS signaling may reinforce MAPK-dependent proliferation, survival, invasion, and mesenchymal transition. In this setting, the CIMP program and NRAS-driven signaling may converge on reduced inflammatory signaling, diminished antigen-presentation capacity, and extracellular matrix remodeling, collectively creating a microenvironment less permissive for effective lymphocyte infiltration.

### Transcriptome-inferred CIMP+ melanomas are associated with reduced benefit from immune checkpoint inhibitors

**Figure 5:**
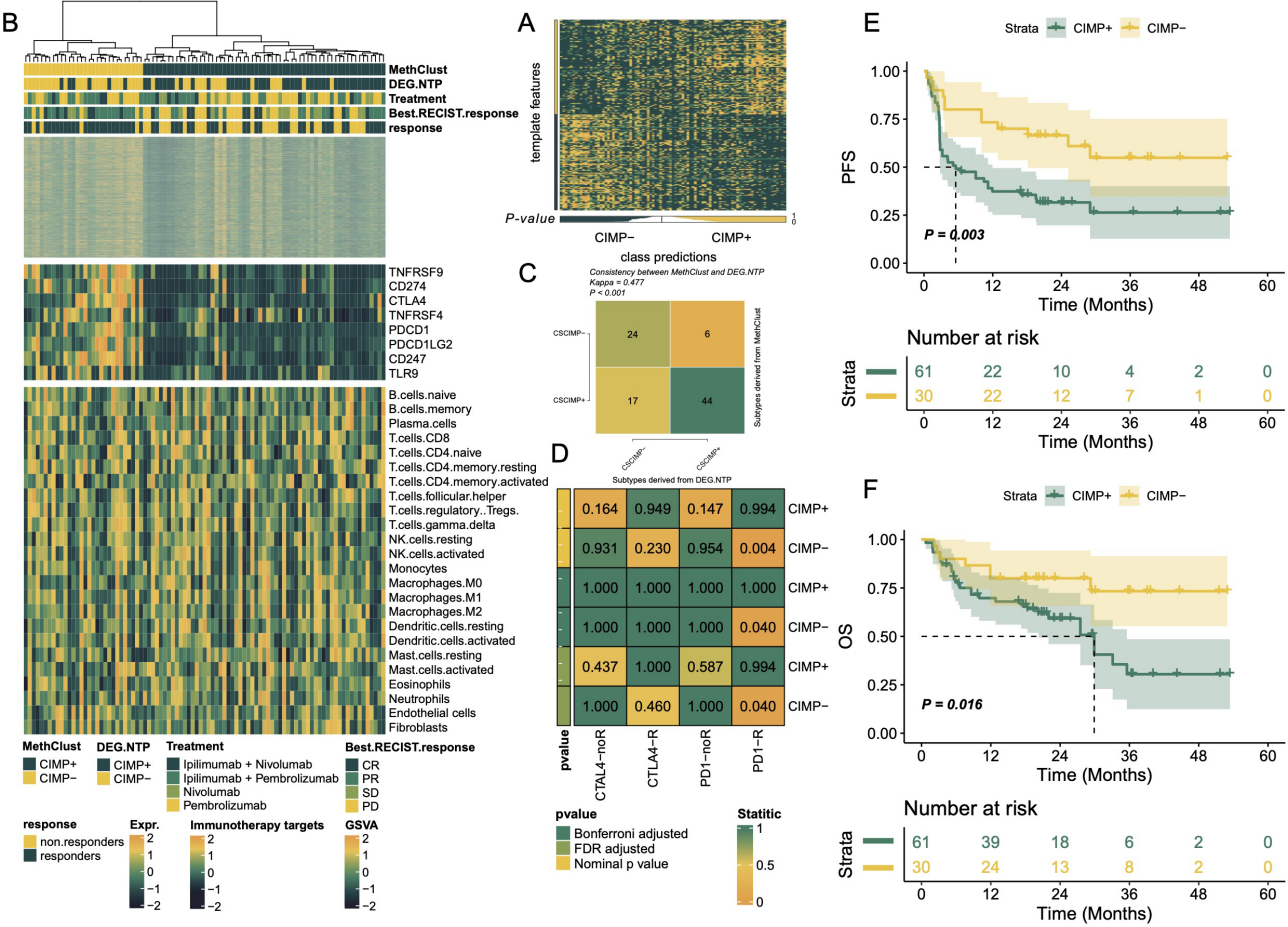
Immunotherapy response and clinical outcomes in CIMP+ melanoma. **(A)** Reclassification of patients from Gide’s cohort based on a 200-gene template, which predicted CIMP+ versus CIMP- status. **(B)** Supervised clustering using 1,707 genes showing promoter hypermethylation-associated reduced expression in the TCGA cohort revealed two subgroups. Heatmap showing differential expression of immune checkpoint genes, immune cell infiltration, and stromal cell infiltration between CIMP+ and CIMP− melanoma subgroups. **(C)** Heatmap displaying the class prediction results for CIMP+ and CIMP- melanoma using two different prediction methods: Meth cluster and DEG.NTP. **(D)** Heatmap displaying the differential response to CTLA-4 and PD-1 inhibition between CIMP+ and CIMP- melanoma subgroups. Statistical significance was assessed using Bonferroni-adjusted p-values, FDR-adjusted p-values, and normal p-values. **(E, F)** In Gide’s cohort, CIMP+ melanoma patients had significantly poorer clinical outcomes, with shorter PFS (P = 0.003) and OS (P = 0.016) compared to CIMP-.

Given the immune-depleted bulk-tumor phenotype of CIMP+ melanomas, we hypothesized that the CIMP-associated expression state might be linked to reduced benefit from immune checkpoint inhibitors (ICIs). We evaluated data from the Gide cohort of metastatic melanoma patients undergoing treatment with anti-PD-1 monotherapy or a combination of anti-PD-1 and anti-CTLA-4 therapy. Since matched methylation data were not available for this cohort, we first generated a 200-gene template using the top 100 significantly and uniquely upregulated genes in each epigenetic subgroup and conducted nearest-template prediction (NTP) to assign each Gide sample to a CIMP-associated transcriptomic state (**Figure 5A**). To further connect the transcriptome and epigenome in this cohort, we additionally performed supervised hierarchical clustering using the 1,707 genes showing promoter hypermethylation-associated reduced expression in the TCGA cohort. This analysis revealed two clusters in which the predicted CIMP+ group showed a generally immune-poor transcriptional profile, whereas the predicted CIMP− group showed stronger immune-infiltrated features (**Figure 5B**). The predicted CIMP+ state was significantly enriched among immunotherapy non-responders compared with the predicted CIMP− state (49.2% vs 13.3%, P < 0.001). NTP and supervised clustering showed high consistency (P < 0.001; **Figure 5C**), supporting the internal robustness of the transcriptomic transfer strategy. In the TCGA cohort, subclass mapping demonstrated that CIMP− exhibited significant transcriptional similarity to a subgroup of melanoma patients responsive to anti-PD1 therapy (adjusted P < 0.05; **Figure 5D**). These findings suggest that the CIMP-associated expression program may help identify patients less likely to benefit from ICI, but they should not be interpreted as direct methylation-based validation of CIMP status in the Gide cohort. The predicted CIMP+ state was associated with significantly poorer prognosis compared with the predicted CIMP− state in Gide’s cohort for both PFS (P = 0.016; **Figure 5E**) and OS (P = 0.003; **Figure 5F**), highlighting its potential clinical relevance in immunotherapy-treated melanoma. Because CIMP status in Gide’s cohort was inferred from transcriptomic data rather than measured by DNA methylation profiling, the observed association should be regarded as evidence that a CIMP-associated expression state is linked to unfavorable ICB outcomes, rather than definitive proof that methylation-defined CIMP alone causes therapeutic resistance.

### CIMP serves as an independent prognostic indicator in cutaneous melanoma

**Figure 6:**
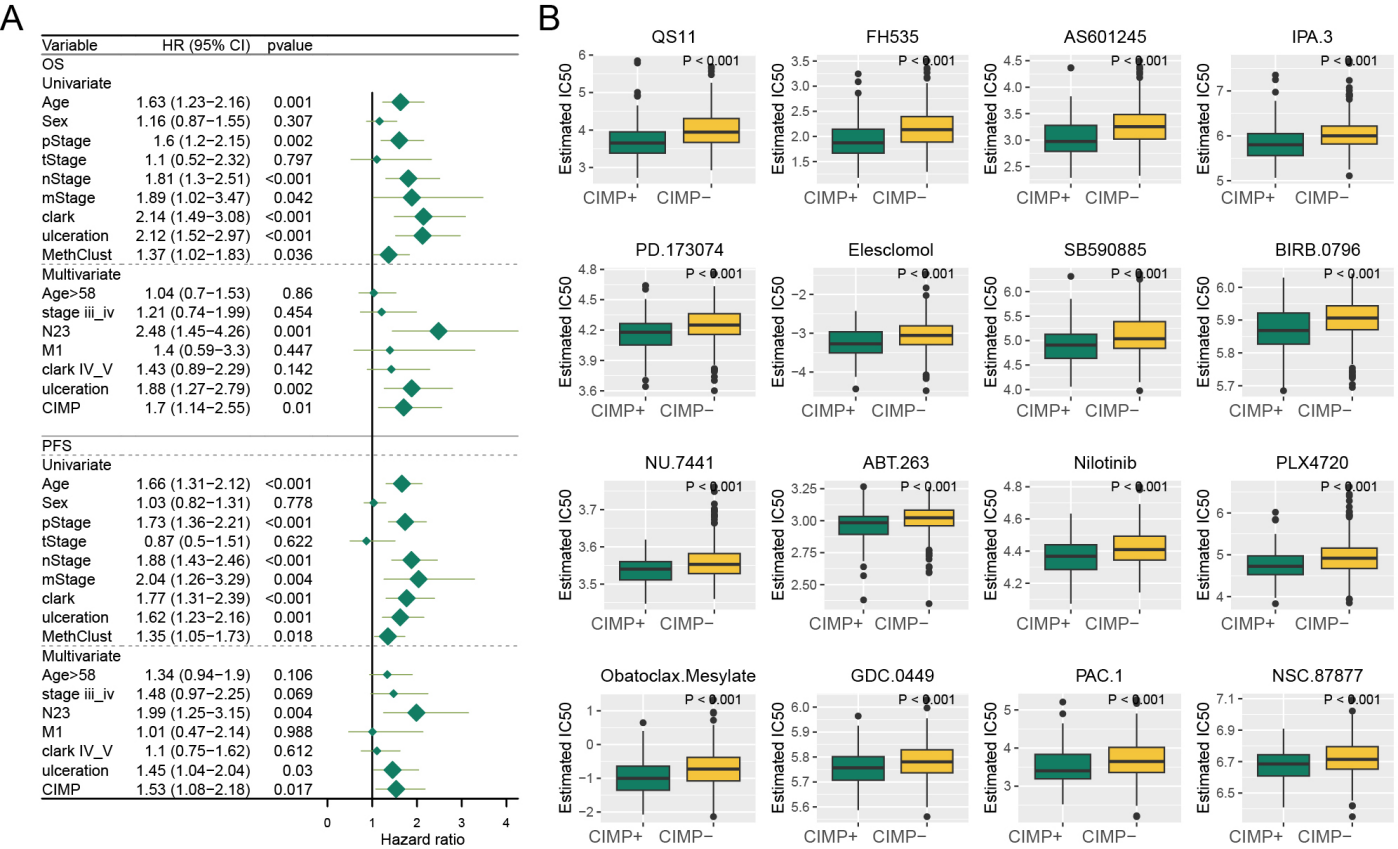
Identification of potential therapeutic targets for CIMP+ melanoma. **(A)** Multivariate Cox proportional hazards regression analysis identified CIMP as an independent prognostic factor for cutaneous melanoma. **(B)** In silico drug screening revealed 16 potential therapeutic agents for treating CIMP+ melanoma, including QS11, FH535, AS601245, and IPA.3, which showed significant efficacy compared to CIMP-.

We then evaluated the potential of CIMP as an independent prognostic indicator for cutaneous melanoma. Cox proportional hazard regression models, both univariate and multivariate, were employed to assess the influence of clinical variables on overall survival (OS) and progression-free survival (PFS). CIMP emerged as a significant independent prognostic factor for overall survival (OS, P = 0.01) and progression-free survival (PFS, P = 0.017), maintaining its significance after adjusting for clinical variables such as age, tumor stage, and ulceration status (**Figure 6A**).

### Identification of potential therapeutic strategies for CIMP+ melanomas/

Given the poor prognosis associated with CIMP+ melanomas, we sought to identify potential therapeutic strategies for these tumors using an in silico drug screening approach. A ridge regression model was constructed to predict drug sensitivity across cutaneous melanoma cell lines, and the predictive model was applied to each of the melanoma cases in the cohort. A total of 16 drugs were identified as potentially effective against CIMP+ melanomas, including QS11, FH535, AS601245, IPA.3, PD.173074, Elesclomol, and BIRB.0796 (all FDR < 0.05; **Figure 6B**; **Supplementary Table 8**). These findings suggest that CIMP+ melanomas may require targeted therapeutic approaches.

### NRAS is enriched in CIMP+ melanoma and promotes aggressive melanoma behavior

**Figure 7:**
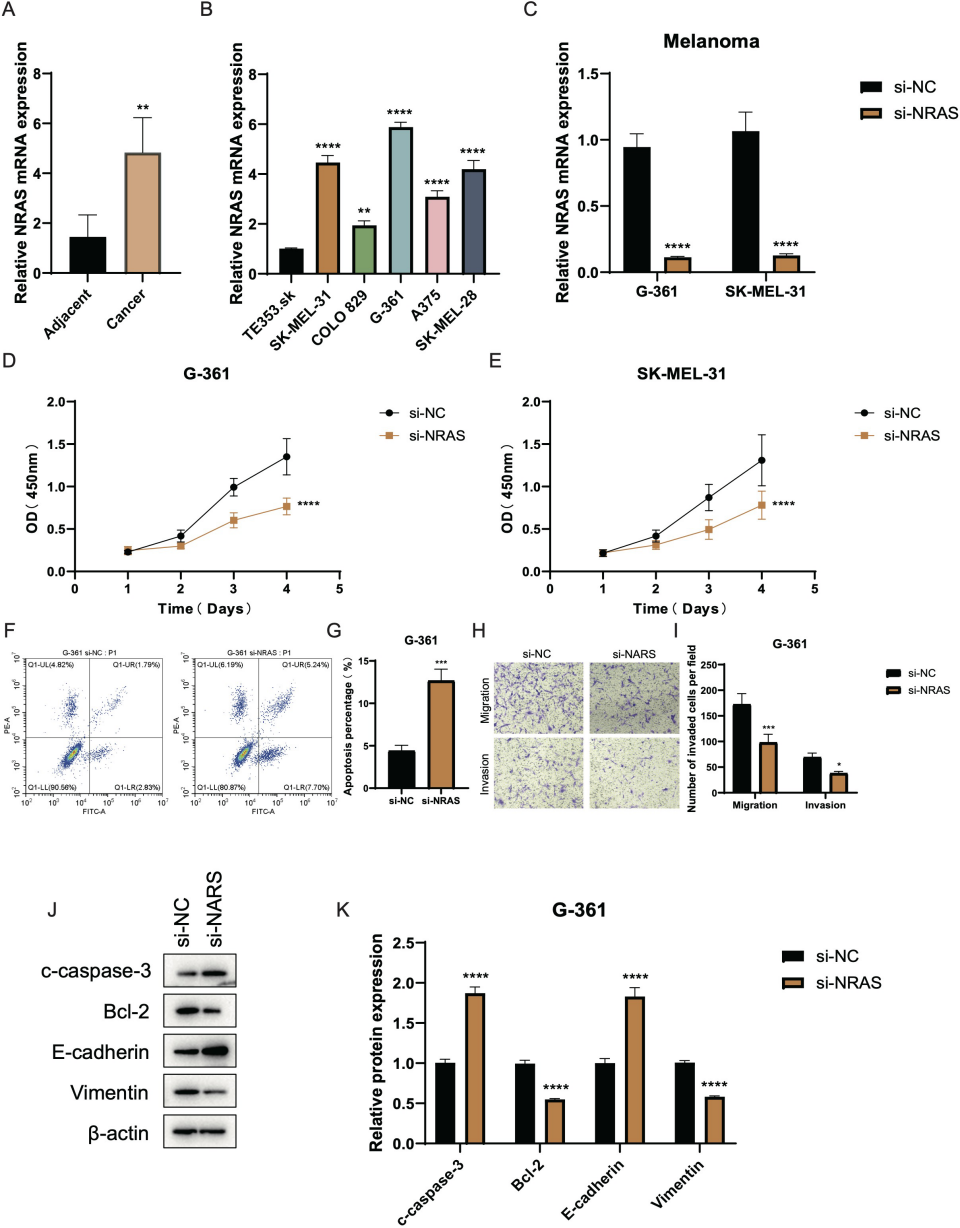
NRAS promotes melanoma cell proliferation, survival, and invasion. **(A)** Relative NRAS mRNA expression in melanoma tissues compared with matched adjacent normal tissues (n = 5 paired samples). **(B)** Baseline NRAS mRNA expression across melanoma cell lines, normalized to the non-tumorigenic TE353.sk cell line. **(C)** Validation of NRAS knockdown efficiency by qRT-PCR in G-361 and SK-MEL-31 cells transfected with si-NRAS or negative control (si-NC).**(D, E)** CCK-8 proliferation assays showing growth curves of G-361 **(D)** and SK-MEL-31 **(E)** cells following NRAS silencing. **(F, G)** Flow cytometry analysis of apoptosis in G-361 cells after transfection. Representative plots **(F)** and quantification of total apoptotic cells **(G–I)** Transwell migration and invasion assays after NRAS knockdown. Representative images and quantitative analysis are shown. **(J, K)** Western blot analysis **(J)** and densitometric quantification **(K)** of cleaved caspase-3, Bcl-2, E-cadherin, and Vimentin following NRAS silencing.

To experimentally validate the functional relevance of NRAS in melanoma progression, we first examined its expression in paired clinical specimens. qRT-PCR analysis revealed that NRAS mRNA levels were significantly elevated in tumor tissues compared with matched adjacent normal tissues (**Figure 7A**). Assessment across melanoma cell lines showed that NRAS expression was highest in G-361 and SK-MEL-31 cells (**Figure 7B**), and these models were therefore selected for loss-of-function studies. Efficient knockdown of NRAS following siRNA transfection was confirmed by qRT-PCR (**Figure 7C**). Functional assays demonstrated that NRAS depletion markedly suppressed cell proliferation in both G-361 and SK-MEL-31 cells over a four-day period, as determined by CCK-8 assays (**Figures 7D, E**). Flow cytometric analysis further showed a significant increase in apoptotic cell populations after NRAS silencing (**Figures 7F, G**). Moreover, Transwell assays revealed that NRAS knockdown substantially impaired both migration and invasion capacities of melanoma cells (**Figures 7H, I**). Consistently, Western blot analysis showed increased cleaved caspase-3 and E-cadherin expression together with decreased Bcl-2 and Vimentin levels following NRAS silencing (**Figures 7J, K**), indicating enhanced apoptosis and suppression of epithelial–mesenchymal transition. Collectively, these data support NRAS as a functional driver of aggressive melanoma cell behavior. However, because these experiments did not interrogate DNA methylation remodeling, promoter-associated repression, immune-cell exclusion, or treatment response, they should be interpreted as evidence for NRAS-associated tumor aggressiveness rather than direct mechanistic proof that NRAS mediates the CIMP phenotype itself.

## Discussion

The clinical challenge posed by melanoma lies in its profound heterogeneity (43), which drives diverse tumor behaviors and variable therapeutic responses. While well-characterized genomic drivers, such as BRAF and NRAS mutations, provide an important framework for understanding melanoma, they fail to fully explain the variability observed in the tumor microenvironment and patient outcomes (44, 45). The study presents the CpG Island Methylator Phenotype (CIMP) as a crucial integrative axis for categorizing cutaneous melanoma. We show that CIMP+ (approximately 25% of cases) is not merely a methylation signature, but instead represents a coherent biological program. CIMP+ is characterized by enrichment of specific genetic alterations, including ARID2 and NRAS mutations, chromosomal instability, promoter hypermethylation associated with reduced expression of immune-related pathways, and an immune-depleted bulk-tumor phenotype associated with poorer survival and unfavorable ICB outcomes. This work highlights CIMP as a critical framework for understanding the genomic, epigenomic, and immunophenotypic heterogeneity in melanoma, offering a pragmatic biomarker to assess clinical aggression and to nominate patients who may require additional treatment strategies beyond standard immunotherapy (46).

The prognostic significance of CIMP+ is evident, operating independently of conventional factors in our multivariable models. In our cohort, the associations with older age and male sex were statistically robust, whereas the observed relationships with more advanced stage and higher mitotic rate did not meet the predefined significance threshold and therefore should be regarded as suggestive trends rather than definitive clinicopathologic correlates (47, 48). Importantly, our finding that CIMP status is independent of sample type (primary versus metastatic) suggests that it represents an intrinsic and relatively stable tumor trait, likely established early in melanoma pathogenesis. This stability stands in contrast to the dynamic, reversible “phenotype switching” observed in melanoma cell states, suggesting that CIMP may represent a more fixed, epigenetically locked program of differentiation blockade. This process may be facilitated by the widespread silencing of polycomb repressor complex 2 target genes, a phenomenon observed both in our study and in others (49).

At the mechanistic level, our data indicate that CIMP+ melanoma arises in a genomic context enriched for both ARID2 and NRAS alterations, but they do not establish a direct causal chain linking NRAS activity to methylation remodeling. The co-enrichment of ARID2 and NRAS mutations is nevertheless noteworthy. ARID2, an essential component of the PBAF chromatin remodeling complex, is often deactivated in melanoma (50). Its loss is not a passive event; studies indicate that ARID2-deficient models exhibit altered DNA damage response and transcriptional regulation (51). It is therefore biologically plausible that chromatin-remodeling defects may create a permissive context for epigenetic dysregulation, while oncogenic NRAS may amplify aggressive phenotypes through MAPK-driven growth and invasion programs. However, the present study did not test whether NRAS activation induces DNA methylation changes, promoter-associated repression, or immune exclusion. We therefore interpret NRAS primarily as a recurrent molecular feature enriched in CIMP+ tumors and as a functionally validated driver of aggressive melanoma behavior, rather than as a proven mediator of the CIMP program. The NRAS-enriched subset may still have important clinical implications, as NRAS-mutant melanoma is notoriously resistant to targeted therapies and shows variable responses to immunotherapy (52). In this context, CIMP+ tumors within the NRAS-mutant subgroup may represent an especially aggressive fraction, but the mechanistic basis for this overlap requires additional investigation. The genomic instability observed in these tumors—evidenced by focal copy-number alterations and chromosomal instability—further supports the existence of a biologically high-risk subset, although the directionality of these relationships remains to be clarified.

A more integrated interpretation is that CIMP+ melanoma may reflect a layered regulatory state rather than a single linear pathway. Chromatin-remodeling defects such as ARID2 mutation could make promoter regions more susceptible to stable epigenetic repression, including repression of genes involved in interferon sensing, antigen processing, and immune-cell recruitment. Concurrently, NRAS/MAPK activation may provide the proliferative and invasive output of this state by promoting survival signaling and mesenchymal-like phenotypes. These two processes are not necessarily causally ordered; instead, they may form a reinforcing circuit in which epigenetic silencing reduces immune visibility, while NRAS-driven tumor-cell plasticity and matrix remodeling reduce the probability of productive T-cell infiltration. This model better explains why CIMP+ tumors show both NRAS enrichment and immune depletion, while avoiding the unsupported conclusion that NRAS alone establishes the CIMP phenotype.

The genetic-epigenetic interplay in CIMP+ tumors is accompanied by a markedly immune-depleted bulk-tumor profile. Our integrative analyzes identified widespread promoter hypermethylation associated with reduced expression of genes involved in interferon-γ signaling and antigen presentation. However, this relationship should be interpreted carefully for two reasons. First, because the CIMP groups were defined on the basis of methylation patterns, the enrichment of hypermethylated loci in CIMP+ partially reflects the underlying classification framework. Second, the reduced expression of immune-related transcripts in bulk tumors may arise from both tumor-cell-intrinsic regulatory repression and diminished immune-cell representation, as supported by the consistently lower immune and stromal infiltration scores in CIMP+ samples (53). We therefore interpret these findings as evidence of methylation-linked immune repression at the bulk-tumor level, rather than definitive proof of direct causal silencing in melanoma cells alone (14). Even with this caveat, the concordance between methylation, transcriptomic, and microenvironmental features supports the view that epigenetic regulation may contribute to the immune-poor state of CIMP+ melanoma. Likewise, the lower immune checkpoint transcripts and GSVA-derived immune signatures observed in CIMP+ are best interpreted as bulk-level correlates of reduced immune/stromal admixture, potentially accompanied by higher tumor purity, rather than as direct evidence for selective suppression of specific immune populations within tumor cells.

This interpretation is also consistent with the observed resistance to immune checkpoint blockade. Checkpoint inhibitors require a pre-existing or therapy-inducible antitumor immune cycle, including antigen presentation, interferon responsiveness, chemokine-driven lymphocyte trafficking, and local T-cell activation. CIMP-associated hypermethylation may interrupt this cycle at several levels by dampening interferon-γ-related transcriptional programs and reducing immune-recruiting signals, whereas NRAS-associated invasive and EMT-like programs may promote a stromal architecture that physically and biochemically limits immune-cell entry. Thus, the CIMP–NRAS association should be viewed as a cooperative framework linking epigenetic immune silencing and oncogenic tumor progression, rather than as evidence for a simple one-directional mechanism.

CIMP+ melanoma was also associated with unfavorable outcomes after ICI therapy, but this conclusion requires moderation. Applying our classifier to transcriptomic data from ICI-treated patients revealed that the transcriptome-inferred CIMP+ state was significantly enriched among non-responders and associated with poorer survival outcomes. These observations support the clinical relevance of the CIMP-associated state and provide a plausible framework for understanding why a subset of patients fails ICI treatment despite the generally immunogenic nature of melanoma. Our findings resonate with previous studies showing that methylation of MHC and immune checkpoint loci can predict poor ICI response (54, 55). However, because direct methylation data were unavailable in the ICB-treated cohort, these results validate the prognostic and predictive relevance of a CIMP-associated expression program rather than the methylation-defined CIMP class itself. In addition, ICB resistance is likely multifactorial; chromosomal instability, impaired antigen processing and presentation, oncogenic MAPK signaling, stromal exclusion, and tumor-purity differences may all contribute to the observed phenotype. Therefore, CIMP-associated methylation should be considered one component of a broader resistance ecosystem rather than the sole driver of immune exclusion or therapeutic failure.

Given the poor prognosis and intrinsic resistance of CIMP+ melanoma to ICI, identifying alternative therapeutic strategies is crucial. Our in silico drug screen identified several promising candidates, including Elesclomol (a pro-oxidative agent) and inhibitors targeting the Wnt pathway. These vulnerabilities may reflect the metabolic and signaling stresses imposed by the CIMP+ state’s rapid evolution and epigenomic dysfunction. This suggests a strategic shift: rather than focusing solely on immunotherapy, patients with CIMP+ melanoma may benefit from targeted therapeutic combinations. Rational treatment strategies could include epigenetic priming with DNA methyltransferase or EZH2 inhibitors to reverse immune silencing, followed by ICI, a concept supported by preclinical models (56–58). Alternatively, targeting the NRAS pathway—such as with the novel BRAF/CRAF inhibitor naporafenib in combination with MEK inhibitors—may be particularly relevant for NRAS-mutant tumors that overlap with the CIMP+ subgroup (59). Co-targeting NRAS-driven signaling and the broader CIMP-associated epigenetic state may therefore merit investigation, although the mechanistic relationship between these processes remains unresolved.

Our study does have limitations. First, the initial probe-selection framework relied on a restricted reference set that combined benign nevi with only two adjacent tissues from TCGA; although this choice was biologically motivated by the need for lineage-matched non-malignant melanocytic comparators, it may have enriched for hypermethylated CpGs and thereby favored identification of a CIMP-like axis. Second, the selection of k = 2 captured the dominant high-/low-methylation split in TCGA, but the three-cluster pattern observed in the external methylation cohort indicates that finer-grained structure may exist and that CIMP should presently be viewed as a robust major axis of methylation stratification rather than a fully exhaustive class system. Third, our external analyzes primarily assessed transferability of a predefined methylation signature and validation of its biological and clinical correlates; they do not replace independent *de novo* subtype discovery with outcome validation in a separate methylation cohort. Fourth, our promoter-centered application of ELMER was intentionally chosen to interrogate proximal methylation–expression coupling, but it does not capture distal enhancer regulation and should not be interpreted as a comprehensive map of regulatory circuitry. Likewise, the observed associations between promoter methylation and reduced transcript abundance do not establish direct causality. Fifth, because the immune-related expression signals were derived from bulk tumor profiles, the inferred repression signatures likely reflect a combination of tumor-intrinsic regulatory changes, reduced immune-cell admixture, and variation in tumor purity. Sixth, the ICB-treated Gide cohort lacked matched methylation profiles; therefore, CIMP status in that cohort was inferred from a TCGA-derived transcriptomic template and should be viewed as a surrogate CIMP-associated expression state. Accordingly, prospective validation in clinically annotated cohorts with pre-treatment methylation profiling remains necessary. The causal relationship between ARID2 loss, CIMP establishment, and immune evasion warrants further functional dissection in appropriate models. Moreover, while bulk analyzes reveal dominant patterns, single-cell multi-omics will be essential to disentangle the cell-type-specific contributions to the CIMP signature and its interaction with stromal cells, such as IL-6-secreting senescent fibroblasts that influence melanoma survival. In addition, we did not perform formal purity-adjusted regression or benchmarking across alternative deconvolution frameworks, and therefore part of the reduced immune-signature signal in CIMP+ may reflect broader differences in tumor cellularity. Importantly, although NRAS knockdown experiments established a role for NRAS in aggressive melanoma cell behavior, we did not test whether NRAS directly regulates DNA methylation patterns, methylation-associated transcriptional repression, immune exclusion, or therapeutic resistance. These mechanistic relationships therefore remain unresolved and require dedicated investigation. In addition, although we clarified the probe-selection rationale, clustering parameters, and transcriptomic transfer procedure, a locked clinical classifier would require more extensive stability analyzes, including bootstrap or consensus clustering, comparison with alternative clustering algorithms and distance metrics, and validation in independent cohorts with paired methylation, transcriptomic, tumor-purity, clinical outcome, and ICB-response data.

Future mechanistic studies should therefore combine methylation editing or DNMT/EZH2 inhibition with NRAS pathway perturbation in melanoma models, followed by assessment of interferon response genes, antigen-presentation molecules, chemokine secretion, and immune-cell killing or co-culture assays. Such experiments would directly test whether reversal of CIMP-associated methylation restores immune visibility and whether NRAS signaling modulates this effect, thereby moving the current association-based model toward causal validation.

In conclusion, CIMP+ melanoma defines a clinically aggressive subtype associated with an immune-depleted bulk-tumor profile and unfavorable outcomes after immune checkpoint blockade. This subtype is characterized by enrichment of specific genetic alterations, including ARID2 and NRAS mutations, widespread chromosomal instability, and promoter hypermethylation linked to reduced immune-related gene expression. Importantly, CIMP+ is independent of conventional clinicopathologic factors, suggesting that it may represent a relatively stable tumor feature established during melanoma progression. These findings highlight the need for therapeutic strategies that consider both methylation-associated immune repression and non-epigenetic resistance mechanisms, including chromosomal instability, antigen-presentation defects, oncogenic signaling, and tumor–stromal remodeling. NRAS appears to mark a particularly aggressive component of this subtype and functionally promotes melanoma cell proliferation, survival, invasion, and epithelial–mesenchymal transition; however, whether NRAS directly contributes to the establishment of the CIMP phenotype, immune exclusion, or therapy resistance remains to be determined. Future studies should validate these findings prospectively in methylation-profiled ICB cohorts, with a focus on functional dissection of ARID2 loss, NRAS-associated epigenetic remodeling, tumor-purity effects, and single-cell tumor–immune interactions in CIMP+ melanoma.

## Data Availability

Publicly available datasets were analyzed in this study. This data can be found here: DNA methylation and transcriptomic profiles from primary and metastatic melanomas were obtained from The Cancer Genome Atlas and validated in an external cohort (GSE120878).Clinical significance was assessed in TCGA and an independent ICB-treated cohort (PRJEB23709).

## Supplementary material

Supplementary Table 1 Clinicopathologic features of cutaneous melanoma stratified by CIMP status. This table lists the clinicopathologic characteristics of cutaneous melanoma patients based on their CIMP (CpG Island Methylator Phenotype) status (CIMP+ vs CIMP-).

Supplementary Table 2 Promoter probes showing differential methylation between CIMP+ and CIMP- subgroups. This table presents the list of 2,510 promoter probes that were significantly differentially methylated between the CIMP+ and CIMP- subgroups in cutaneous melanoma.

Supplementary Table 3 Genes associated with differentially methylated promoter probes in CIMP+ and CIMP- subgroups. This table lists the 84,448 promoter-gene pairs associated with 2,510 differentially methylated probes from **Supplementary Table 2**.

Supplementary Table 4 Complete list of genes associated with promoter hypermethylation and reduced expression in CIMP+ melanoma. This table lists the 1,707 genes associated with promoter hypermethylation and reduced expression in CIMP+ melanoma. These genes are derived from the 8,448 promoter-gene pairs identified in **Supplementary Table 3**.

Supplementary Table 5 Gene ontology and pathway enrichment of genes associated with promoter hypermethylation and reduced expression in CIMP+ melanoma.This table displays the enrichment analysis results for the genes listed in **Supplementary Table 4**.

Supplementary Table 6 Mutational landscape of cutaneous melanoma: differential mutation rates in CIMP+ and CIMP-. This table presents the mutational landscape of 894 genes in cutaneous melanoma, with differential mutation rates between CIMP+ and CIMP- subgroups.

Supplementary Table 7 Mutual exclusivity and co-occurrence of mutations in CIMP+ and CIMP- melanoma. This table presents the analysis of mutual exclusivity and co-occurrence of mutations in key melanoma-related genes, including BRAF, NRAS, ARID2, and CDKN2A, across the CIMP+ and CIMP- subgroups.

Supplementary Table 8 Predicted drug sensitivity in CIMP+ melanoma using in-silico screening. This table lists the results of an in-silico drug screening approach applied to cutaneous melanoma cases with CIMP status.
